# Transferring Desirable Genes from *Agropyron cristatum* 7P Chromosome into Common Wheat

**DOI:** 10.1371/journal.pone.0159577

**Published:** 2016-07-26

**Authors:** Mingjie Lu, Yuqing Lu, Huanhuan Li, Cuili Pan, Yong Guo, Jinpeng Zhang, Xinming Yang, Xiuquan Li, Weihua Liu, Lihui Li

**Affiliations:** National Key Facility for Crop Gene Resources and Genetic Improvement, Institute of Crop Science, Chinese Academy of Agricultural Sciences, Beijing 100081, China; Institute of Genetics and Developmental Biology, CHINA

## Abstract

Wheat-*Agropyron cristatum* 7P disomic addition line Ⅱ-5-1, derived from the distant hybridization between *A*. *cristatum* (2*n* = 4*x* = 28, PPPP) and the common wheat cv. Fukuhokomugi (Fukuho), displays numerous desirable agronomic traits, including enhanced thousand-grain weight, smaller flag leaf, and enhanced tolerance to drought. In order to transfer these traits into common wheat, Ⅱ-5-1 was induced by ^60^Co-γ ray, leading to the creation of 18 translocation lines and three deletion lines. Genomic *in situ* hybridization (GISH) and fluorescence *in situ* hybridization (FISH) indicated that multiple wheat chromosomes were involved in the translocation events, including chromosome 2A, 3A, 5A, 7A, 3B, 5B, 7B, 3D and 7D. *A*. *cristatum* 7P chromosome was divided into 15 chromosomal bins with fifty-five sequence-tagged site (STS) markers specific to *A*. *cristatum* 7P chromosome. Seven and eight chromosomal bins were located on 7PS and 7PL, respectively. The above-mentioned translocation and deletion lines each contained different, yet overlapping 7P chromosomal fragments, covering the entire *A*. *cristatum* 7P chromosome. Three translocation lines (7PT-13, 7PT-14 and 7PT-17) and three deletion lines (del-1, del-2 and del-3), which contained the common chromosomal bins 7PS1-3, displayed higher thousand-grain weigh than Fukuho, suggesting that potential genes conferring high thousand-grain weigh might be located on these chromosomal bins. Therefore, wheat-*A*. *cristatum* 7P translocation lines with elite traits will be useful as novel germplasms for wheat genetic improvement.

## Introduction

Bread wheat (*Triticum aestivum* L.), serving as a major source of calories, is one of the most important crops in the world. However, genetic improvement of wheat for high yield and quality to meet the demand from an ever-growing population has become challenging, as wheat breeding has been bottlenecked by a decreasing genetic diversity [1,2]. Therefore, researchers are persistently seeking for novel germplasms with broad genetic diversity for wheat genetic improvement.

One effective approach to create novel germplasms is to transfer desirable genes conferring superior agronomic traits into common wheat from its wild relatives. This approach has been successfully employed in wheat improvement. For example, yield-related genes located on *Th*. *bessarabicum* chromosome arm 2JS were transferred into common wheat through wheat-*Th*. *bessarabicum* translocation line T2JS-2BS·2BL [3]. The genes conferring longer spikes and more kernels, as well as the seed storage protein genes conferring positive effect on bread making quality, were transferred into common wheat from *Dasypyrum villosum*, respectively [4–5]. Two new wheat-*Thinopyrum ponticum* translocation lines RWG33 and RWG34 were found to carry *Sr43*, which was resistant to the Ug99 race complex [6]. Stem rust resistance genes *SrTA10187* and *SrTA10171* were both derived from *Aegilops tauschii* [7]. Two T1BL·1RS translocation lines derived from the crosses between common wheat cultivar and rye, showed high stripe rust resistance and good quality [8]. Taken together, a large number of desirable genes have been successfully transferred into common wheat, resulting in a plenty of novel wheat germplasm. Thus far, several translocation lines have been widely used in wheat breading, and the two typical examples were wheat-rye 1BL·1RS and wheat-*Haynaldia villosa* 6VS·6AL translocation lines [9–11].

*Agropyron cristatum* (L.) Gaertn. (2*n* = 4*x* = 28, PPPP), one of the most important wild relatives of wheat, harbours multiple desirable agronomic traits, such as enhanced grain number per spike, multiple floret per spikelet, resistance to diseases, and tolerance to abiotic stresses [12–15]. In order to exploit these traits and transfer them into common wheat, distance hybridization between the common wheat cv. Fukuhokomugi (Fukuho) and *A*. *cristatum* was carried out in 1990s [16–19]. F_1_ hybrids were obtained successfully, and then a series of wheat-*A*. *cristatum* disomic addition lines were acquired [20–22]. These wheat-*A*. *cristatum* disomic addition lines were backcrossed with Fukuho or self-pollinated for several generations, until all of them displayed stable agronomic traits. Thus far, several wheat-*A*. *cristatum* disomic addition lines have been induced, producing various translocation, substitution, deletion and introgression lines [15,23–25]. For example, wheat-*A*. *cristatum* 6P addition line 4844–12 was used as the fundamental material to produce numerous wheat-*A*. *cristatum* 6P derivatives, some of which contain genes conferring enhanced grain number per spike [15,26], enhanced fertile tiller number per plant [23]. It’s also reported that wheat-*A*. *cristatum* disomic 2P addition line II-9-3 was induced, producing various wheat-*A*. *cristatum* 2P derivatives with high resistance to powdery mildew [27].

The wheat-*A*. *cristatum* disomic 7P addition line Ⅱ-5 was found to display small flag leaf, enhanced thousand-grain weight, tolerance to drought. In order to transfer these elite traits into common wheat, Ⅱ-5-1 was irradiated with ^60^Co-γ. Various wheat-*A*. *cristatum* 7P derivatives were acquired, Among them 18 translocation lines and three deletion lines were studied in this project. Cytological and molecular methods were applied to figure out the genetic constitutions of the 21 lines; The *A*. *cristatum* 7P chromosome was divided into different chromosomal bins, constructing the physical map of *A*. *cristatum* 7P chromosome. Besides, the agronomic traits, especially spike traits, were evaluated, and the chromosomal bins with thousand-grain weight-controlling genes were determined. Therefore, this study will not only provide novel germplasm for wheat breeding but also help to understand the characterization of *A*. *cristatum* 7P chromosome.

## Materials and Methods

### Plant materials

The wheat-*A*. *cristatum* 7P disomic addition line Ⅱ-5-1 (2*n* = 44) was derived from the wide hybridization between *A*. *cristatum* accession Z559 (2*n* = 4*x* = 28, PPPP) and *Triticum aestivum* cv. Fukuhokomugi (Fukuho) (2*n* = 6*x* = 42, AABBDD).

### Irradiation of wheat-*A*. *cristatum* 7P disomic addition line II-5-1

The II-5-1 plants were irradiated with ^60^Co-γ ray at a dosage of 20 Gray (Gy) and a dose rate of 0.5 Gy/min, when the plants were at the booting stage [28]. All the irradiated plants were artificially emasculated and pollinated with fresh pollens from the recipient parent Fukuho. F_1_ seeds were harvested, and then planted to produce the BC_1_F_1_ and BC_2_F_1_ populations.

### Morphological indexes of II-5-1 at the germination stage

One hundred seeds from II-5-1 and Fukuho, respectively, were placed on filter paper with an osmotic potential of -0.5Mpa induced by the polyethylene glycol solution (PEG-6000) as described by Michael and Kaufman [29] and Gholamin et al. [30]. Mock treatment was conducted by using water. Each experiment was conducted three times with three replicates each time. All Petri dishes were placed in a growth chamber for seven days at a temperature of 22 ± 2°C and 50% relative humidity. On the seventh day, multiple morphological indices including germination rate, germination index, coleoptile length, radicle number were recorded from 20 seedlings in each replication. Some morphological indices were calculated using the following formulas:

Germination rate = Total number of seeds germinated / Total number of seeds used x 100%

Relative germination rate = Germination rate in PEG / Germination rate in water

Germination index was defined as:

Germination index = 1.0*n1+6/7*n2+5/7*n3+4/7*n4+3/7*n5+2/7*n6+1/7*n7, where n1-n7 were germination rates from the first day to the seventh day.

### GISH and FISH analysis

Genomic *in situ* hybridization (GISH) was performed to detect *A*. *cristatum* 7P chromosomal segments of Ⅱ-5-1 as well as its derivative lines. The root tips of all the materials were harvested when they grew to about 2 cm, and the chromosomes were prepared as described by Song et al. [28]. Genomic DNA of *A*. *cristatum* and Fukuho was isolated by CTAB method [31], and they were used as probe and block, respectively. *A*. *cristatum* genomic DNA was labelled with digoxigenin-11-dUTP. Meiosis of Ⅱ-5-1 were studied as described by Jauhar and Peterson [32]. Briefly, young spikes of Ⅱ-5-1 at the pollen mother cells (PMCs) metaphase I (MI) stage were fixed in Carnoy’s solution (6-ethanol: 3-chloroform: 1-acetic acid) for 24 h and stored at 4°C until use. GISH was performed on the root tip cells and PMCs as described by Liu et al. [33].

To identify the translocated wheat chromosomes, fluorescence *in situ* hybridization (FISH) was employed using repetitive DNA clones *pAs1* and *pHvG39* as probes, respectively [34]. *A*. *cristatum* genomic DNA and the clone *pAs1* were labeled by the Digoxigenin-Nick Translation Mix, while the clone *pHvG39* was labeled by Biotin-Nick Translation Mix. All the hybridization signals were observed using an OLYMPUS AX80 fluorescence microscope (Olympus Optical Co., Tokyo, Japan) and captured with a CCD camera (Diagnostic Instruments, Sterling Heights, MI, USA).

### Construction of the physical map of 7P chromosome with STS markers

Sequence-Tagged Site (STS) markers were developed based on *A*. *cristatum* transcriptome sequences, which were deposited in the NCBI Transcriptome Shotgun Assembly (TSA) archive under the accession number GBAU00000000 [35]. These STS markers were used to characterize *A*. *cristatum* chromosome 7P, and then physically mapped on the different chromosomal bins. *A*. *cristatum* and Fukuho were used as the positive and negative control, respectively. The PCR programme was one cycle at 94°C for 5 min, followed by 36 cycles at 94°C for 1 min, at annealing temperature for 1 min, and at 72°C for 1 min, with a final extension at 72°C for 10 min. The amplified PCR products were separated by polyacrylamide gel electrophoresis (PAGE) with an acrylamide concentration of 8% and stained with silver.

### Evaluation of agronomic traits

Plant materials were planted in 2.0 m rows, spaced 30 cm apart in the Beijing experiment station. The agronomic traits evaluated included the spike traits (grain number per spike, spikelet per spike, thousand-grain weight, grain length and grain width), the size of flag leaf, and also resistance to drought stress. The spike traits were measured as previously described [23]. Flag leaf length and width were measured at the milk stage. Drought tolerance evaluation was carried out as previously described [36]. Briefly, Ⅱ-5-1 and Fukuho were treated with 20% Polyethylene glycol-6000 (PEG) after culturing for 7 days in water, and PEG-induced phenotypes were scored after 48 h.

## Results

### Characteristics of the wheat-*A*. *cristatum* 7P disomic addition line Ⅱ-5-1

**Fig 1:**
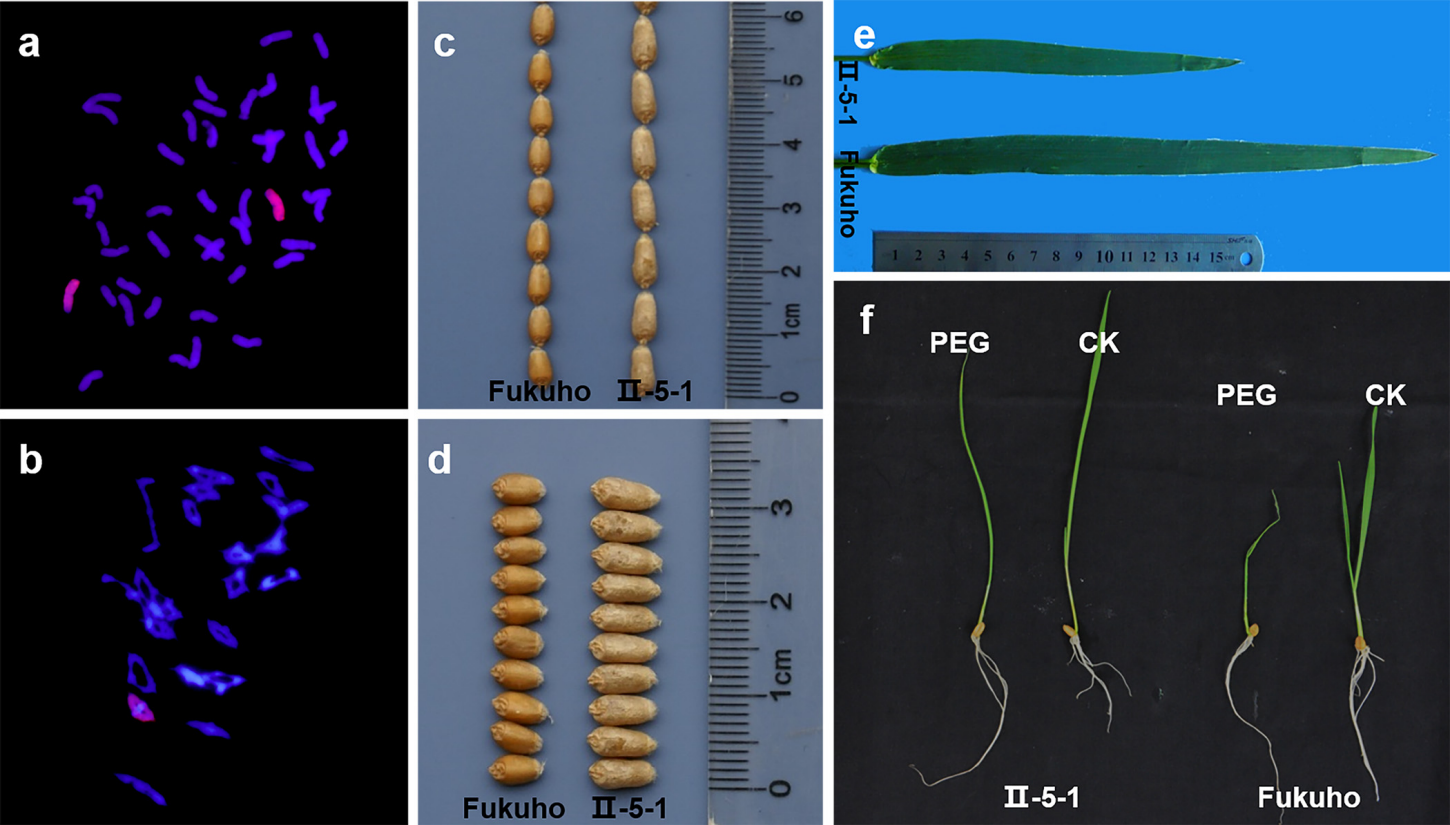
Identification of the wheat-*A*. *cristatum* disomic addition lineⅡ-5-1. a, GISH pattern of II-5-1 showing 42 wheat chromosomes (shown in blue) and two *A*. *cristatum* chromosomes (shown in green and pointed by arrows); b, 21 bivalents are shown at the PMCs metaphase I (MI) stage of II-5-1; c-d, The morphologies of grain length (c) and width (d) of Ⅱ-5-1 and Fukuho; e, The morphology of flag leaf of Ⅱ-5-1 and Fukuho; f, The phenotype of seedling induced by PEG of Ⅱ-5-1 and Fukuho.

Ⅱ-5-1 was derived from the wide hybridization between *A*. *cristatum* and Fukuho, followed by self-pollination for six generations. GISH result showed that there were 44 chromosomes, including 42 wheat chromosomes and two *A*. *cristatum* chromosomes (Fig 1A). Chromosomal pairing behavior in the pollen mother cells (PMCs) of II-5-1 was observed, and the chromosomal configuration was 2*n* = 22 II; Besides, neither trivalents nor quadrivalents were observed (Fig 1B), suggesting that II-5-1 was a stable disomic addition line.

II-5-1 displayed stable agronomic traits under three years’ field observation. As shown in Table 1, slight difference in plant height was observed between II-5-1 and Fukuho, but there was no significant difference in fertile tiller number per plant and spike length. Ⅱ-5-1 showed enhanced thousand-grain weight and grain length compared with its recurrent parent Fukuho. The grain number per spike of Ⅱ-5-1 was lower than that of Fukuho, and there was no significant difference in grain width and spikelet numbers per spike. The morphologies of grain length and grain width in Ⅱ-5-1 and Fukuho were shown in Fig 1C and 1D. As shown in Fig 1E and Table 1, Ⅱ-5-1 possessed narrower and shorter flag leaf compared with that of Fukuho. As shown in Table 2, relative germination rate, germination index, coleoptile length, radicle number of II-5-1 were higher than those of Fukuho, and relative germination index, relative coleoptile length, relative radicle number of II-5-1 were also increased compared with Fukuho, respectively. At the seedling stage, Ⅱ-5-1 displayed less severe wilting than Fukuho (Fig 1F). These results suggested that Ⅱ-5-1 displayed higher tolerance to drought compared with Fukuho. In sum, we propose that multiple elite agronomic traits in II-5-1, such as enhanced thousand-grain weight, longer grain length and higher tolerance to drought might be attributed to desirable alien genes from the *A*. *cristatum* 7P chromosome.

**Table 1:** Agronomic traits of wheat-*A*. *cristatum* addition line Ⅱ-5-1 and Fukuho.

Materials	Thousand-grain weight	Grain length	Grain width	Grain numbers per spike	Spikelet numbers per spike	Flag leaf length	Flag leaf width	Plantheight	Fertile tiller numbers per plant	Spike length
**Ⅱ-5-1**	**45.6±0.5a**	**7.84±0.05a**	**3.17±0.04a**	**30.6±2.3b**	**17.9±0.7a**	**17.3±1.9b**	**1.37±0.08b**	**105.0±2.3a**	**20.5±2.0a**	**11.1±1.2a**
**Fukuho**	**38.4±2.3b**	**6.65±0.10b**	**3.21±0.08a**	**63.5±5.0a**	**18.5±1.0a**	**24.6±2.5a**	**1.78±0.18a**	**93.0±3.7b**	**21.3±3.6a**	**10.6±0.9a**

Significant differences in the mean are indicated at the *P* < 0.05 (lowercase letters), based on T tests.

**Table 2:** Morphological indices of II-5-1 and Fukuho induced by PEG at the germination stage.

	Relative Germination Rate	Germination Index	Relative Germination Index	Coleoptile Length	Relative Coleoptile Length	Radicle Number	Relative Radicle Number
Ⅱ-5-1	0.94	1.89±0.01a	0.85	23.4±1.1a	0.84	4.1±0.7a	0.95
Fukuho	0.88	1.83±0.01b	0.75	19.5±1.9b	0.71	3.3±0.5b	0.60

Significant differences in the mean are indicated at the *P* < 0.05 (lowercase letters), based on T tests.

### Cytological identification of wheat-*A*. *cristatum* 7P translocation and deletion lines

In order to integrate the elite traits of Ⅱ-5-1 into common wheat, wheat-*A*. *cristatum* 7P addition lineⅡ-5-1 was irradiated with ^60^Co-γ ray, and then backcrossed with the recipient parent Fukuho. Then BC_1_F_1_ seeds harvested from the irradiated plants were backcrossed with Fukuho to obtain BC_2_F_1_ plants. 219 BC_1_F_1_ lines were acquired totally, among which 18 translocation lines and three deletion lines were further investigated by GISH and FISH.

**Fig 2:**
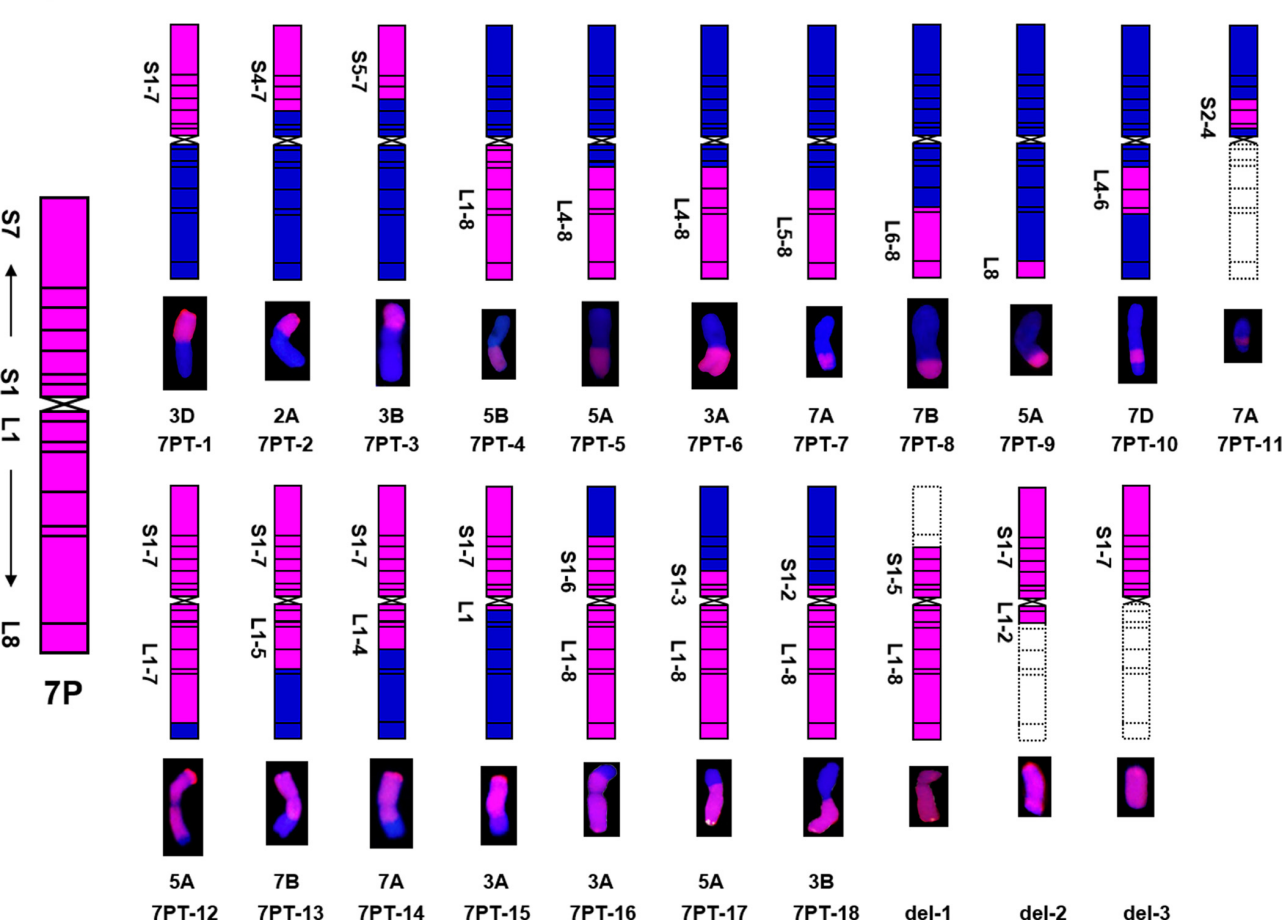
Eighteen wheat-*A*.*cristatum* translocation lines and three deletion lines were identified. The left map showing the schematic diagram of *A*. *cristatum* chromosome 7P. Pink and blue color represented *A*. *cristatum* 7P and wheat chromosomal segments, respectively. Dotted line boxes indicated the deleted chromosomal segments of *A*. *cristatum* 7P chromosome.

As shown in Table 3 and Fig 2, 18 translocation lines contained 42 chromosomes consisting of 41 wheat chromosomes, while three deletion lines contained 43 chromosomes consisting of 42 wheat chromosomes. 18 translocation lines were categorized into four translocation types, according to the relative size of the translocated chromosomal segments compared to either 7P chromosome arm (Fig 2 and Table 3). These four types were as follows: whole-arm translocation, large segmental translocation, small segmental translocation and intercalary translocation.

**Table 3:** 18 wheat-*A*. *cristatum* 7P translocation lines and three deletion lines were acquired.

Materials	Chromosome number	Translocated wheat chromosomes	Translocation types	7P chromosome bins	Generations
7PT-1	42	3D	7PS·3DL	S1-7	BC_1_F_1_
7PT-2	42	2A	7PS-2AS·2AL	S4-7	BC_1_F_1_
7PT-3	42	3B	7PS-3BS·3BL	S5-7	BC_1_F_1_
7PT-4	42	5B	5BL·7PL	L1-8	BC_1_F_1_
7PT-5	42	5A	5AS·5AL-7PL	L4-8	BC_1_F_1_
7PT-6	42	3A	3AS·3AL-7PL	L4-8	BC_1_F_1_
7PT-7	42	7A	7AS·7AL-7PL	L5-8	BC_1_F_1_
7PT-8	42	7B	7BS·7BL-7PL	L6-8	BC_1_F_1_
7PT-9	42	5A	5AS·5AL-7PL	L8	BC_1_F_1_
7PT-10	42	7D	7DS·7DL-7PL-7DL	L4-6	BC_1_F_1_
7PT-11	42	7A	7AS-7PS-7AS	S2-4	BC_1_F_1_
7PT-12	42	5A	7PS·7PL-5AL	S1-7, L1-7	BC_1_F_1_
7PT-13	42	7B	7PS·7PL-7BL	S1-7, L1-5	BC_1_F_1_
7PT-14	42	7A	7PS·7PL-7AL	S1-7, L1-4	BC_1_F_1_
7PT-15	42	3A	7PS·7PL-3AL	S1-7, L1	BC_1_F_1_
7PT-16	42	3A	3AS-7PS·7PL	S1-6, L1-8	BC_1_F_1_
7PT-17	42	5A	5AS-7PS·7PL	S1-3, L1-8	BC_1_F_1_
7PT-18	42	3B	3BS-7PS·7PL	S1-2, L1-8	BC_1_F_1_
del-1	43	N/A	N/A	S1-5, L1-8	BC_1_F_1_
del-2	43	N/A	N/A	S1-7, L1-2	BC_1_F_1_
del-3	43	N/A	N/A	S1-7	BC_1_F_1_

N/A: Not applicable

**Fig 3:**
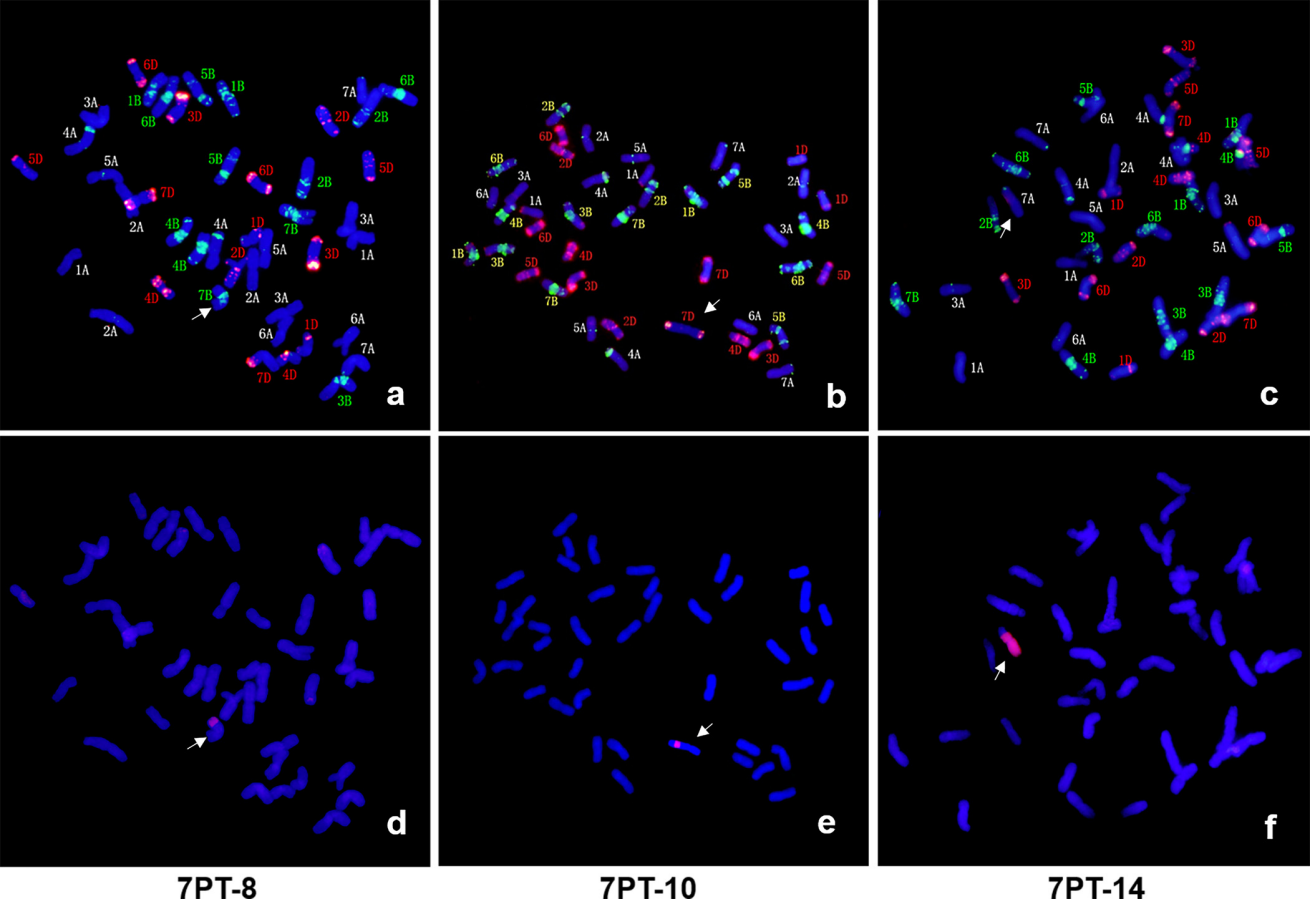
Three wheat-*A*. *cristatum* translocation lines identified by GISH and FISH. 7PT-8 was the small chromosomal segment translocation line, which was translocated to wheat chromosome 7B (Fig 3A and 3D). 7PT-10 was the small chromosomal segment translocation line, which was intercalated into wheat chromosome 7D (Fig 3B and 3E). 7PT-14 was the large chromosomal segment translocation line, which was translocated to wheat chromosome 7A (Fig 3C and 3F). FISH patterns were shown in a-c, in which the clone *pHvG39* and *pAs1* was labeled green and red, respectively. GISH patterns were shown in d-f, in which *A*. *cristatum* and wheat DNA was stained red and blue, respectively.

7PT-1 and 7PT-4 were both whole-arm translocation lines, in which translocation occurred to wheat chromosomes 3D and 5B, respectively. Seven small segmental translocation lines were identified (7PT-2, 7PT-6, 7PT-5, 7PT-9, 7PT-7, 7PT-3 and 7PT-8), in which translocation occurred on wheat chromosomes 2A, 3A, 5A, 5A, 7A, 3B and 7B, respectively. There were seven large segmental translocation lines (7PT-15, 7PT-12, 7PT-17, 7PT-14, 7PT-18, 7PT-16 and 7PT-13), which were translocated to 3A, 5A, 5A, 7A, 3B, 3A and 7B, respectively; 7PT-10 and 7PT-11 belonged to intercalary translocation lines, which were translocated to wheat chromosome 7DL and 7AS, respectively; However, only the short arm was left in 7PT-11, and the long arm was missing. GISH and FISH patterns of three translocation lines (7PT-8, 7PT-10 and 7PT-14) were shown in Fig 3, and GISH and FISH patterns of other translocation lines were shown in S1 Fig. Beside 18 translocation lines, there were three deletion lines (del-1, del-2 and del-3) identified. There was only one chromosome arm left in del-3, and the other chromosome arm was missing. There were partial chromosomal segments less than one chromosome arm missing in del-1 and del-2.

### Construction of the physical map of 7P chromosome

**Fig 4:**
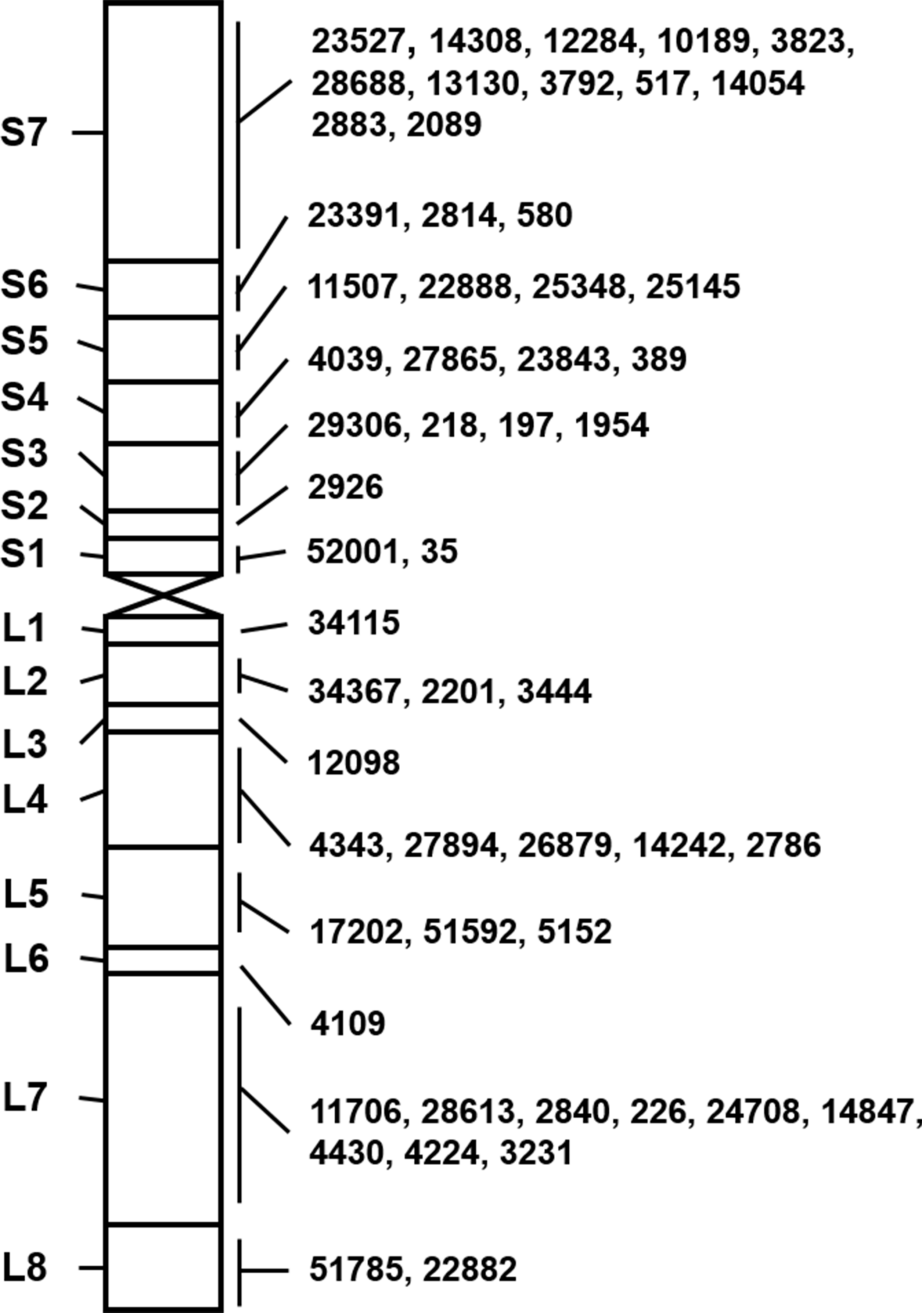
The physical map of *A*. *cristatum* 7P chromosome with 55 STS markers. Left panel: 15 chromosomal bins of *A*. *cristatum* 7P chromosome; Right panel: Physical locations of STS markers specific to 7P chromosome. The words "*Agc*" in front of the STS markers are omitted.

**Fig 5:**
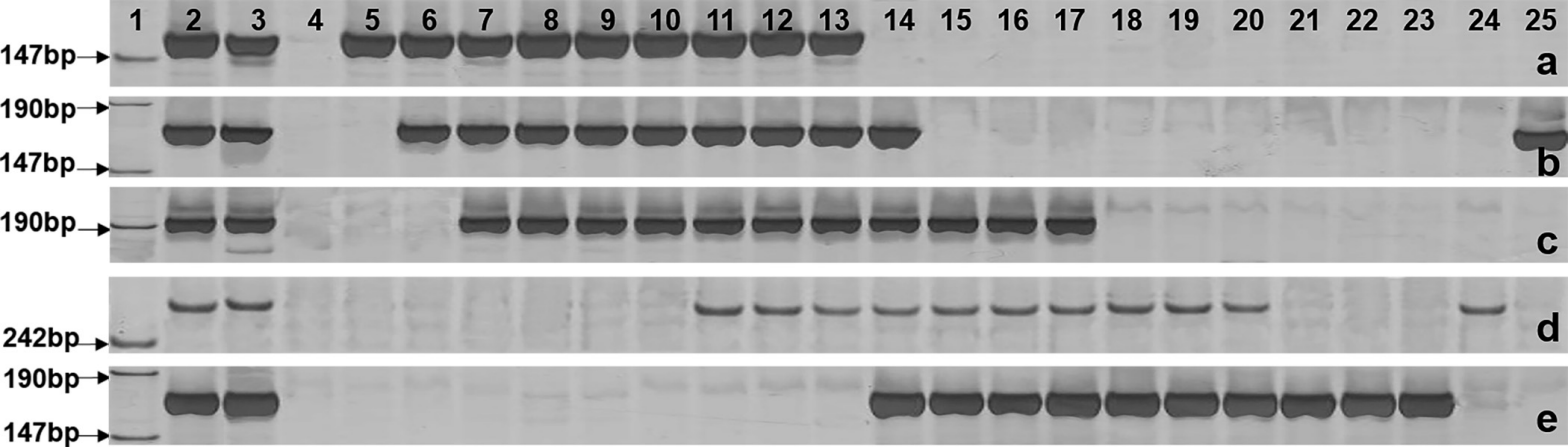
PCR amplification patterns of five STS markers. a, *Agc23527*; b, *Agc389*; c, *Agc52001*; d, *Agc27894*; e, *Agc51785*. 1, Marker; 2, Z559; 3, Ⅱ-5-1; 4, Fukuho; 5, 7PT-3; 6, 7PT-2; 7, 7PT-1; 8, del-3; 9, 7PT-15; 10, del-2; 11, 7PT-14; 12, 7PT-13; 13, 7PT-12; 14, 7PT-16; 15, del-1; 16, 7PT-17; 17, 7PT-18; 18, 7PT-4; 19, 7PT-5; 20, 7PT-6; 21, 7PT-7; 22, 7PT-8; 23, 7PT-9; 24, 7PT-10; 25, 7PT-11.

We further constructed the physical map of *A*. *cristatum* 7P chromosome with STS markers anchored to each bin. A large number of STS markers were screened to find those that can successfully be amplified in both *A*. *cristatum* and Ⅱ-5-1, and in at least one of the 21 translocation and deletion lines, but not in Fukuho, resulting in the discovery of 126 7P-specific STS markers. Among them, 55 STS markers displayed high amplification efficiency and reproducibility, and were chosen for downstream analysis (S1 Table). According to the co-occurrences of the STS markers and 7P chromosomal bins in these translocation and deletion lines, the 55 STS markers were mapped to different bins of *A*. *cristatum* chromosome 7P, with 30 and 25 STS markers located on 7PS and 7PL, respectively (Fig 4). The chromosomal bins of 7PS and 7PL near the centromere were named as 7PS1 and 7PL1, respectively; while the chromosomal bins near the ends were named as 7PS7 and 7PL8, respectively (Fig 4). Each chromosomal bin contained different STS markers, ranging from 1 to 12. The primer sequences of 55 STS markers were listed in S2 Table, and PCR amplification patterns of five markers were shown as examples in Fig 5.

As shown in Fig 2 and Table 3, 18 translocation lines contained different chromosomal bins of 7PS and 7PL. Four translocation lines (7PT-1, 7PT-2, 7PT-3 and 7PT-11) only contained both 7PS or part of 7PS, and seven translocation lines (7PT-4, 7PT-5, 7PT-6, 7PT-7, 7PT-8, 7PT-9 and 7PT-10) contained both 7PL or part of 7PL. Four translocation lines (7PT-12, 7PT-13, 7PT-14 and 7PT-15) contained 7PS and part of 7PL, while three translocation lines (7PT-16, 7PT-17 and 7PT-18) contained 7PL and part of 7PS. The deletion line del-1 included the entire 7PL arm and 7PS1-5, del-2 included the entire 7PS and 7PL1-2, and del-3 only included the entire 7PS. From these results, we could infer that all the 7P chromosomal bins have been translocated onto wheat chromosomes. All these results indicated that various *A*. *cristatum* 7P chromosomal bins were translocated onto 18 translocation lines and three deletion lines, and all these chromosomal bins can cover the entire *A*. *cristatum* 7P chromosome.

### Evaluation of spike traits of wheat-*A*. *cristatum* 7P translocation and deletion lines

**Fig 6:**
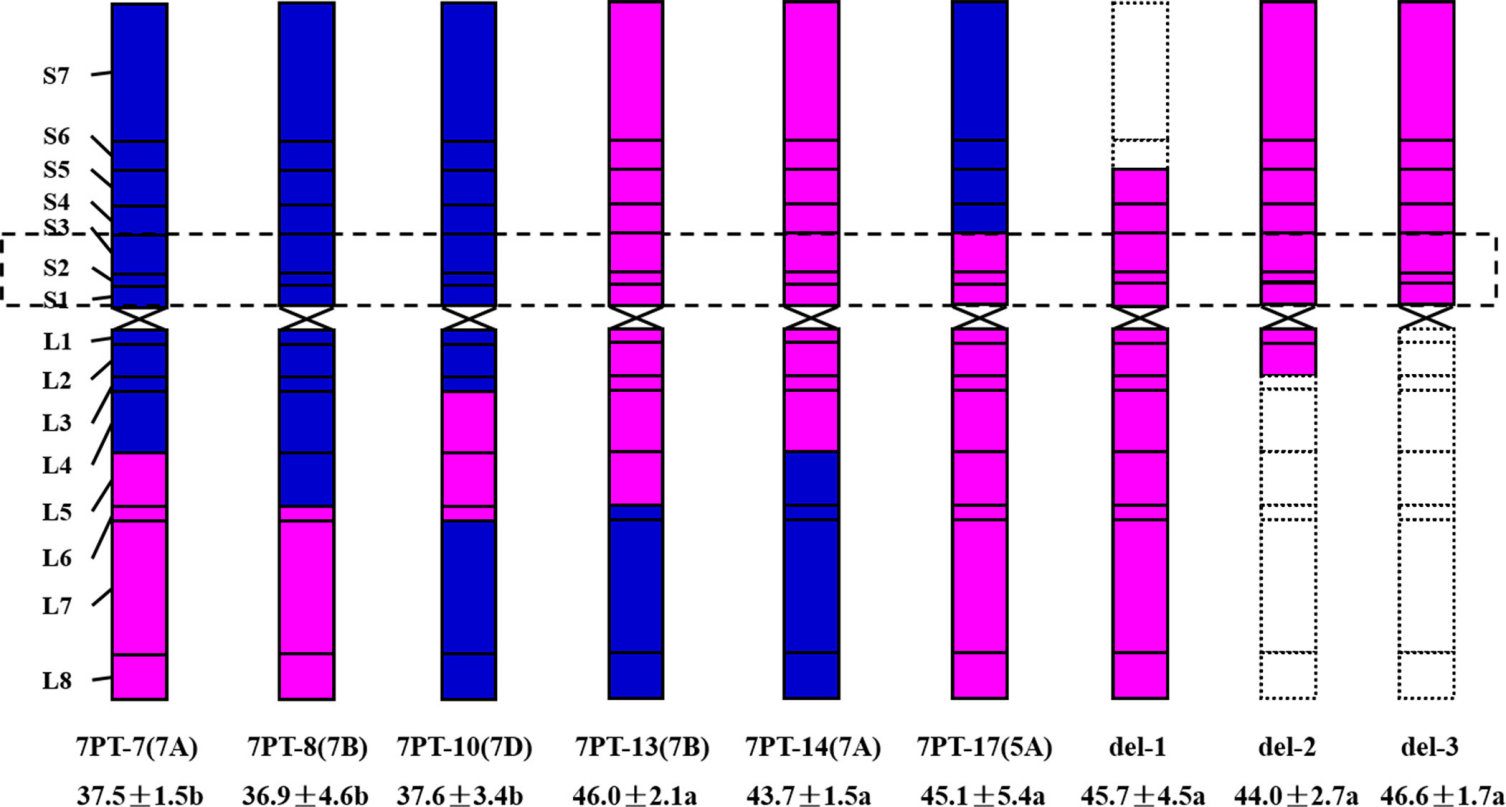
Six lines sharing one common chromosomal bins 7PS1-3 displayed high thousand-grain weight.

Agronomic traits, especially the spike traits, were investigated in six translocation (7PT-7, 7PT-8, 7PT-10, 7PT-13, 7PT-14 and 7PT-17) and three deletion lines (del-1, del-2 and del-3) at the BC_2_F_1_ generations. As shown in Table 4, three translcoation lines (7PT-13, 7PT-14 and 7PT-17) as well as three deletion lines displayed higher thousand-grain weight and longer grain length than those of Fukuho, respectively. However, there were no significant difference in both thousand-grain weight and grain length among other three translocation lines (7PT-7, 7PT-8 and 7PT-10) and Fukuho. There was no significant difference in grain width in all the nine lines as well as two parents. Besides, all the nine lines except 7PT-7 showed less grain number per spike than that of Fukuho, but some of them showed higher grain number per spike than that of II-5-1. There was no significant difference in spikelet number per spike between two parents (II-5-1 and Fukuho), but all the nine lines except 7PT-7 and 7PT-10 contained more spikelet number per spike than that of either parent. As shown in Fig 6, the chromosomal bins 7PS1-3 were present in three translocation lines (7PT-13, 7PT-14 and 7PT-17) and three deletion lines but absent in other three translocation lines. All the results suggested that there might be genes conferring high thousand-grain weight and grain length on the chromosome bins 7PS1-3 of *A*. *cristatum* chromosome.

**Table 4:** Evaluation of the spike traits of some wheat-*A*. *cristatum* translocation and deletion lines.

Materials	Translocation types	Thousand-grain weight	Grain length	Grain width	Grain number per spike	Spikelet numberper spike
**Ⅱ-5-1**	N/A	45.6±0.5a	7.84±0.05a	3.17±0.04a	30.6±2.3d	17.9±0.7d
**Fukuho**	N/A	38.4±2.3b	6.65±0.10d	3.21±0.08a	63.5±5.0a	18.5±1.0d
**7PT-7**	7AS·7AL-7PL	37.5±1.5b	6.74±0.08d	3.17±0.13a	60.5±7.8a	19.5±2.1bcd
**7PT-8**	7BS·7BL-7PL	36.9±4.6b	6.69±0.25d	3.28±0.08a	35.3±4.1d	20.8±1.3abc
**7PT-10**	7DS·7DL-7PL-7DL	37.6±3.4b	6.75±0.08d	3.34±0.08a	37.7±7.5cd	19.0±2.6cd
**7PT-13**	7PS·7PL-7BL	46.0±2.1a	7.38±0.08bc	3.27±0.17a	29.7±4.5d	20.7±0.6abc
**7PT-14**	7PS·7PL-7AL	43.7±1.5a	7.23±0.03bc	3.36±0.21a	50.5±7.8b	21.5±1.4ab
**7PT-17**	5AS-7PS·7PL	45.1±5.4a	7.33±0.04bc	3.35±0.20a	38.0±6.8cd	21.0±1.4abc
**del-1**	N/A	45.7±4.5a	7.21±0.11c	3.30±0.21a	46.3±9.3bc	20.9±1.0abc
**del-2**	N/A	44.0±2.7a	7.41±0.14b	3.27±0.19a	37.0±7.5cd	21.7±2.1ab
**del-3**	N/A	46.6±1.7a	7.26±0.20bc	3.42±0.19a	48.5±4.9b	22.0±1.4a

Significant differences in the mean are indicated at the *P* < 0.05 (lowercase letters), based on Duncan’s multiple range tests.

## Discussion

### Various translocation lines were produced by ionizing radiation

There are large quantities of elite genes in the wild relatives of wheat that can be used to improve the yield, quality, disease resistance and stress tolerance in wheat breeding. Generally speaking, creation of wheat alien addition lines is the first key step to transfer these desirable genes into common wheat. However, wheat alien addition line could not be used directly due to its large alien chromosomal segments. Therefore, wheat alien addition lines are often used as the fundamental materials to produce translocation lines with small alien chromosomal segments.

There were several methods to induce addition lines, such as gamma irradiation, CS *ph1b* system and gametocidal chromosome. Considering the low level of pairing between *A*. *cristatum* P genome and wheat ABD genomes, development of translocation lines by ionizing radiation seems to be a more appropriate approach [37]. Compared with other approaches, there’re much more chromosomal breakages by using this method, resulting in various translocation types [2]. In this study, 18 different wheat-*A*. *cristatum* 7P translocation lines and three deletion lines were obtained, which was mainly attributed to multiple chromosomal breakages caused by ^60^Co-γ irradiation. Besides, two intercalary translocation lines (7PT-10 and 7PT-11) were caused by double-strand chromosomal breakages. Therefore, ionizing radiation proved to be an effective way to produce translocation lines in this study. The effectiveness of gamma irradiation was also illustrated by several other examples. Various 6P and 2P translocation lines were produced from the wheat-*A*. *cristatum* 6P addition line 4844–12 [28] and 2P addition line II-9-3 by ^60^Co-γ irradiation [27], respectively. A series of structural aberrations involving *Thinopyrum bessarabicum* chromosome 4J were obtained through gamma radiation [38]. Plenty of wheat-*Haynaldia villosa* translocation lines with *Pm21* were acquired by ionizing radiation [39].

### Establishment of the physical map of 7P chromosome with STS markers

Cytological methods such as GISH/FISH are often used to identify alien chromosomal segments. However, these methods are ineffective in identifying small chromosomal segments. Moreover, it is not feasible to screen a large number of translocation lines by GISH/FISH, due to the fact that chromosome preparation and hybridization is quite labor-intensive and time-consuming. Compared with the cytological method, molecular marker is more effective to detect alien chromosomal segments. After genotyping the translocation line with molecular markers, we will get a general idea of the translocated chromosomal segments quickly.

In this study, 18 different translocation lines and three deletion lines were characterized by GISH/FISH and 55 STS markers. *A*. *cristatum* 7P chromosome were divided into 15 chromosomal bins depending on the presence or absence of STS markers, leading to the construction of the physical map of 7P chromosome. Once the physical map of 7P chromosome was constructed, genes from this chromosome could be easily located on specific chromosomal bins. In this study, genes conferring enhanecd thousand-grain weight and grain length were preliminarily mapped on the chromosomal bins 7PS1-3. There’re other examples reported previously, showing the constructed physical maps with novel alien genes. For example, the physical map of *Thinopyrum bessarabicum* chromosome 4J consisted of 24 segmental blocks, and the blue-grained gene *BaThb* was mapped on the block 4JL-11 [38]. The physical map of *A*. *cristatum* 6P was built with 255 STS markers, and the leaf rust resistance genes were mapped on the region 6PS-0.81–1.00 [24].

### Wheat-*A*. *cristatum* 7P translocation lines are potentially valuable in wheat breeding

Grain number per spike, fertile tiller number per plant and thousand-grain weight are three main factors determining wheat yield. Among these three factors, thousand-grain weight is least affected by environment [40]. Therefore, it’s important to increase thousand-grain weight in order to stably enhance the wheat yield. Thousand-grain weight is mainly determined by grain width, length and thickness [41–44]. In this study, wheat-*A*. *cristatum* 7P addition line Ⅱ-5-1 displayed enhanced thousand-grain weight and drought tolerance, and also a small flag leaf. It has been reported that a small flag leaf lead to higher photosynthetic rate of the whole plant and in turn higher grain weight [45,46]. A small flag leaf was also reported to reduce transpiration and enhance drought tolerance [47]. Whether the small size of the flag leaf in Ⅱ-5-1 contributed to its high grain weight and drought tolerance remains to be investigated.

Among the 18 translocation lines reported in this study, six translocation lines (7PT-7, 7PT-8, 7PT-10, 7PT-13, 7PT-14 and 7PT-17) and three deletion lines (del-1, del-2 and del-3) displayed good agronomic performance (especially seeds-setting). According to FISH results, the translocations in all six translocation lines except 7PT-17 occurred on the wheat chromosomes which belonged to wheat homoeologous group 7. These results suggested that wheat chromosome 5A, as well as 7B and 7A, conferred good homoeologous compensation with *A*. *cristatum* chromosome 7P. The good homoeologous compensation between 7P and 5A might be attributed by the genetic rearrangement of *A*. *cristatum* chromosome 7P, and the similar phenomenon was also observed on *A*. *cristatum* chromosome 6P [48]. Three translocation lines and three deletion lines with chromosomal bins 7PS1-3 showed increased thousand-grain weight compared with Fukuho, while three translocation lines without chromosomal bins 7PS1-3 displayed decreased thousand-grain weight. The results suggested that there may be some genes conferring high thousand-grain weight on 7PS1-3. However, the agronomic traits of other translocation lines couldn’t be evaluated due to their bad seeds-setting, which might be caused by irradiation or poor compensation. The specific chromosomal locations and identities of superior genes on the chromosomal bins 7PS1-3 conferring high thousand-grain weight still need further investigation. Therefore, to recover the genetic compensation and effectively utilize these lines in future breeding programs, we need to further reduce the linkage drag, preferably by back-crossing or *ph1b* system.

## Supporting Information

S1 Fig Fifteen wheat-*A*. *cristatum* translocation lines were identified by GISH and FISH. FISH patterns were shown in a1-o1, while GISH patterns were shown in a2-o2. a, 7PT-1; b, 7PT-2; c, 7PT-3; d, 7PT-4; e, 7PT-5; f, 7PT-6; g, 7PT-7; h, 7PT-9; i, 7PT-11; j, 7PT-12; k, 7PT-13; l, 7PT-15; m, 7PT-16; n, 7PT-17; o, 7PT-18.
(PDF)

S1 Table The PCR amplification results of 55 STS markers in all the materials. (XLS)

S2 Table The primers sequences of 55 STS markers. (XLS)
