# Supplementary figures and images for: Transferring Desirable Genes from Agropyron cristatum 7P Chromosome into Common Wheat
Source: PLoS One. 2016 Jul 26;11(7):e0159577. doi: 10.1371/journal.pone.0159577 (PMC4961395; doi:10.1371/journal.pone.0159577)

Fig. S1

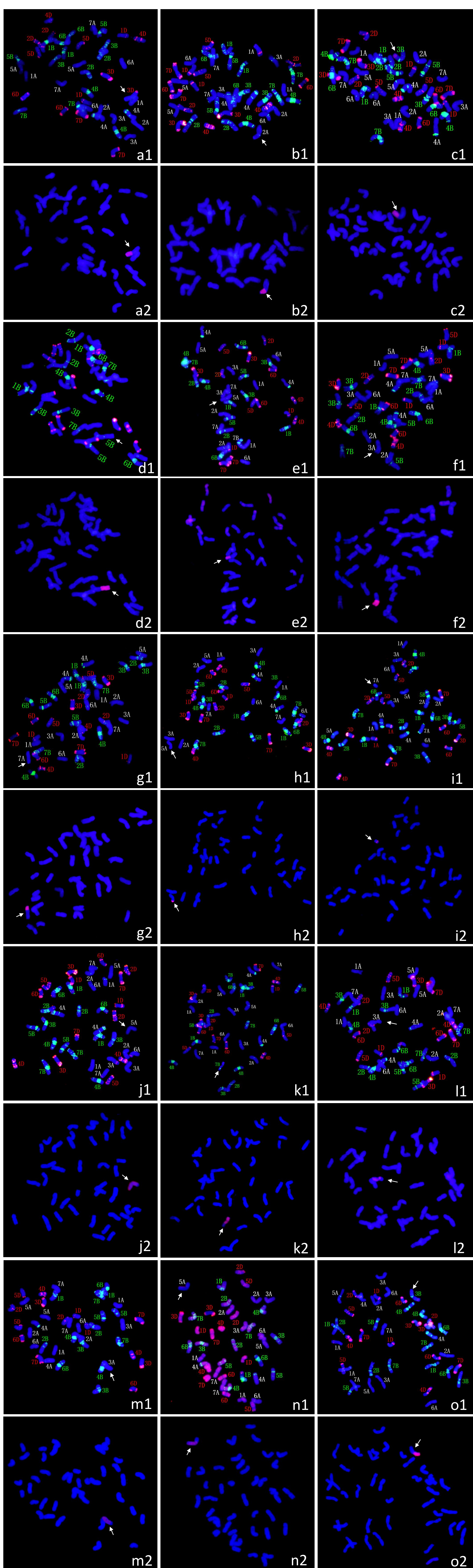

Supplement: S1 Fig — FISH patterns were shown in a1-o1, while GISH patterns were shown in a2-o2. a, 7PT-1; b, 7PT-2; c, 7PT-3; d, 7PT-4; e, 7PT-5; f, 7PT-6; g, 7PT-7; h, 7PT-9; i, 7PT-11; j, 7PT-12; k, 7PT-13; l, 7PT-15; m, 7PT-16; n, 7PT-17; o, 7PT-18. (PDF) [file pone.0159577.s001.pdf]
